# Emergence of ON1 genotype of human respiratory syncytial virus subgroup A in China between 2011 and 2015

**DOI:** 10.1038/s41598-017-04824-0

**Published:** 2017-07-14

**Authors:** Jinhua Song, Yan Zhang, Huiling Wang, Jing Shi, Liwei Sun, Xiaojie Zhang, Zifeng Yang, Wenda Guan, Hong Zhang, Pengbo Yu, Zhengde Xie, Aili Cui, Teresa I. Ng, Wenbo Xu

**Affiliations:** 1 WHO WPRO Regional Reference Measles/Rubella Laboratory and Key Laboratory of Medical Virology Ministry of Health, National Institute for Viral Disease Control and Prevention, China Center for Disease Control and Prevention, Beijing, People’s Republic of China; 2 Lu Juan Community Health Center of Daxing Region, Beijing, People’s Republic of China; 3 Jilin Children’s Medical Center Children’s Hospital of Changchun, Changchun, People’s Republic of China; 4 State Key Laboratory of Respiratory Disease, National Clinical Research Center for Respiratory Disease, First Affiliated Hospital of Guangzhou Medical University, Guangzhou Guangdong, People’s Republic of China; 5 Hunan Provincial Centers for Disease Control and Prevention, Changsha, People’s Republic of China; 6 Shaanxi Provincial Centers for Disease Control and Prevention, Xian, People’s Republic of China; 7 Beijing Children’s Hospital, Capital Medical University, Beijing, People’s Republic of China; 8 AbbVie, Inc., North Chicago, IL USA; 9 Medical College, Anhui University of Science & Technology, Huainan, People’s Republic of China

## Abstract

A molecular epidemiological study of human respiratory syncytial virus (HRSV) was conducted to examine the distribution of its subgroups and genotypes, as well as to identify its transmission pattern in China. A total of 705 samples collected from 9 provinces in China between January 2008 and February 2015 were identified as HRSV-positive and were subsequently sequenced. Of these, 336 samples were HRSV subgroup A (HRSVA), 368 samples were HRSV subgroup B (HRSVB), and 1 sample contained both HRSVA and HRSVB. These 705 HRSV sequences, together with 766 HRSV sequences downloaded from GenBank, were analyzed to understand the recent circulation patterns of HRSV in China. HRSVB predominated in the 2008/2009 and 2009/2010 seasons, whereas HRSVA predominated in the 2010/2011 and 2011/2012 seasons; HRSVA and HRSVB co-circulated during 2012/2013 and 2014/2015. Phylogenetic analysis showed most of the HRSVA sequences clustered into 2 genotypes, namely, NA1 and ON1. The ON1 genotype was first detected in China in 2011, and it quickly replaced the NA1 genotype to become the most prevalent HRSVA genotype circulating in China between 2013 and 2015. Continuous epidemiological surveillance and molecular characterization of HRSV should be conducted to monitor the evolution of HRSV in China.

## Introduction

Human respiratory syncytial virus (HRSV) is one of the leading causes of acute respiratory tract infection in young children ( ≤ 5 years old), the elderly or immunocompromised patients^1–5^. In 2005, about 33.8 million new episodes of HRSV-associated acute lower respiratory infection (ALRI) occurred worldwide in children younger than 5 years of age. Among these patients, approximately 66,000–199,000 died from the infection, with 99% of these deaths occurring in developing countries^6^. In China, a study of 28,369 patients with ALRI from 81 (out of 108) sentinel hospitals in 22 provinces showed that the most frequently detected virus was HRSV (9.9%), and HRSV was the most common etiology (17.0%) in children younger than 2 years of age^7^. In New York City, HRSV infection was a major cause for hospitalization due to respiratory infection for patients with age < 4 or ≥ 75 years old; these patients may need additional control measures to mitigate the risk of infection during HRSV seasons^8^. Annual infection rate of HRSV in the USA ranged from 2% to 10% in community-dwelling older adults, and 5% to 10% in older adults living in congregate settings^9^. Therefore, HRSV-associated lower respiratory tract infection is a big concern for public health.

HRSV is a member of the *Pneumovirinae* subfamily in the *Paramyxoviridae* family. It consists of a non-segmented, single-strand negative RNA genome packaged in a lipid envelope. The genome of HRSV is about 15.2 kb in length and encodes 10 genes and 11 proteins: NS1, NS2, N, P, M, SH, G, F, M2-1, M2-2, and L^10^. The F protein and G protein are the major surface glycoproteins. Both of them can stimulate the production of neutralizing antibodies. The sequence of the F protein is highly conserved, while that of the G protein is hypervariable. Due to the genetic diversity in the G gene, sequence encoding the second hypervariable region in the C-terminal (HVR2) of the G protein has been used for genotyping of HRSV. According to the genetic characteristic and reactivity with monoclonal antibodies, HRSV is classified into 2 subgroups, A (HRSVA) and B (HRSVB)^11^. To date, there are 15 genotypes of HRSVA (GA1-GA7, SAA1, CB-A, NA1-4 and ON1-2)^12–15^, and 24 genotypes of HRSVB (GB1-GB4, SAB1-SAB4, URU1-2, BA1-12, GB5/CB1 and CBB)^16–20^. The HRSVA genotype ON1 was first detected with a 72-nucleotide duplication in the HVR2 region encoded by the G gene in Canada in 2010. Moreover, a lineage of the ON1 genotype was identified as a new genotype (ON2) in Italy in 2015^21, 22^.

In this study, we collected and sequenced HRSV-positive samples collected from 9 sentinel hospitals in 9 provinces in China between 2008 and 2015. In addition, HRSV sequences from 5 additional Chinese provinces were downloaded from Genbank on February 2016 and analyzed together with the sequences we generated. The molecular epidemiology of HRSVA in 14 provinces in China was analyzed and the characteristics of these HRSVA sequences were examined.

## Results

### HRSV samples

In this study, 705 out of 4246 (16.60%) samples collected between January 2008 and February 2015 from 9 hospitals located in Beijing, Hebei, Jilin, Shandong, Shaanxi, Gansu, Shanghai, Guangdong, and Hunan provinces in China were tested positive for HRSV using double-channel real-time reverse transcription-polymerase chain reaction (RT-PCR)^23^. Of these, 336 (47.66%) samples were identified as HRSVA, 368 (52.20%) samples were identified as HRSVB and 1 (0.14%) sample identified as both HRSVA and HRSVB. Genetic sequences of all of the HRSVA samples were generated and used for genotyping and sequence analysis. Additionally, 766 Chinese HRSV sequences with available sample collection information were downloaded from GenBank and included in the sequence analysis. Distribution of HRSV samples collected in China from 2008 to 2015 by year and geographical region is shown in Table 1.

**Table 1:** Distribution of HRSV samples collected in China from 2008 to 2015 by year and geographical region.

	Province/region of sample collection														
Year	Beijing	Gansu	Guangdong	Zhejiang	Hebei	Hunan	Shaanxi	Shanghai	Sichuan	Tibet	Hong Kong	Jilin	Chongqing	Shandong	Total
2008	36 (14)	31(0)	—	1 (0)	—	—	—	6 (0)	—	—	6 (0)	—	18 (0)	—	98 (14)
2009	159 (112)	6 (1)	2 (2)	2 (0)	—	—	—	19 (0)	—	—	6 (0)	1 (1)	16 (0)	—	211 (116)
2010	68 (28)	9 (9)	12 (12)	—	2 (2)	—	39 (39)	34 (5)	16 (0)	—	14(0)	—	45 (0)	—	239 (95)
2011	85 (1)	21 (21)	37 (27)	—	—	1 (1)	5 (5)	12 (0)	—	7 (0)	13 (0)	—	60 (0)	—	241 (55)
2012	61 (0)	—	10 (1)	30 (0)	—	25 (25)	0	10 (0)	—	—	1 (0)	12 (12)	36 (0)	—	185 38)
2013	27 (0)	—	8 (0)	24 (0)	4 (4)	15 (15)	36 (36)	—	—	—	—	77 (77)	14 (0)	—	205 (132)
2014	65 (28)	—	—	—	2 (2)	23 (23)	13 (13)	—	—	—	—	122 (122)	—	18 (18)	243 (206)
2015	—	—	—	—	—	—	—	—	—	—	—	49 (49)	—	—	49 (49)
Total	501 (183)	67 (31)	69 (42)	57 (0)	8 (8)	64 (64)	93 (93)	81 (5)	16 (0)	7 (0)	40 (0)	261 (261)	189 (0)	18 (18)	1471 (705)

Numbers shown are the sum of sequences collected in this study and those downloaded from GenBank; sequences collected in this study are shown within parentheses.

### Clinical characteristic of patients with HRSVA infection

This study focused mainly on the analysis of data from the HRSVA samples. Of the 337 HRSVA patients from whom samples were collected and sequenced in this study, clinical information were available for 242 (71.81%) of them. Most of the 242 samples (228; 94.21%) were collected from inpatients, and the rest (14; 5.8%) were from outpatients. The age of the patients ranged from 1 day to 75 years old, with median age being 2.28 years old. Most of the patients were less than 4 years old (92.15%) and the majority of the patients (53.11%) were less than 1 year old. The male to female ratio was 2.13:1 (68.05% vs 31.95%). Of the 242 patients, 172 (71.07%) were diagnosed with lower-respiratory tract infection, including 45 (26.16%) with pneumonia, 13 (7.56%) with asthmatic bronchitis with pneumonia, 114 (66.28%) with other lower-respiratory tract infections. Moreover, 7 (2.90%) patients were diagnosed with upper-respiratory tract infection, while 63 (26.03%) patients were diagnosed with other diseases (3 cases of enteritis, 2 cases of mycoplasma pneumonia, 2 cases of laryngitis, 1 case of aplastic anemia, 1 case of asthma and 54 cases with unknown diagnosis). There was no significant difference (χ^2^ = 7.736, P = 0.117) between the males and the females in the number of each of the 3 major HRSVA genotypes (NA1, ON1 and NA3) detected, but there was statistically significant difference (χ^2^ = 9.334, p = 0.009) between the two age groups (≤ 2 vs. > 2 years old) in the number of each of these 3 genotypes detected: NA1 (77 vs. 19), ON1 (57 vs. 35), and NA3 (36 vs. 9). Therefore, more NA1, ON1 and NA3 samples were identified in patients who were younger than 2 years of age.

### Phylogenetic analysis of HRSVA sequences

**Figure 1:**
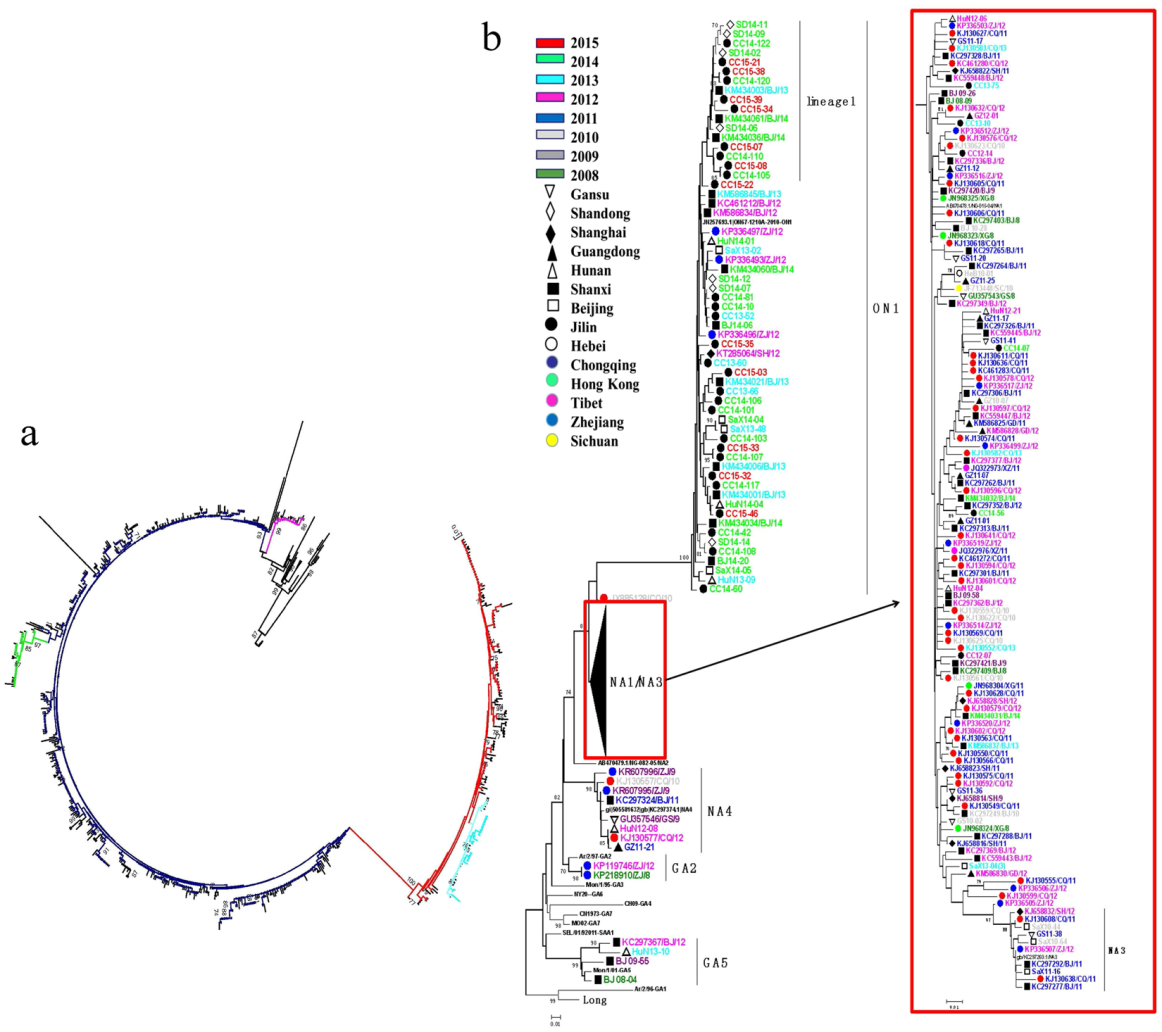
(**a**) Phylogenetic tree of HRSVA sequences from 14 provinces in China between January 2008 and February 2015. The branch of the ON1 genotype is colored in red, lineage 1 is colored in light fluorescence blue, the NA3 genotype is colored in green, the NA4 genotype is colored in purple, and the NA1 genotype is colored in navy. (**b**) Phylogenetic tree of representative HRSVA sequences obtained from January 2008 to February 2015 in China. Representative sequences were chosen from those with the pairwise distances > 0.025. Years of sample collection are indicated by the colors of the sequence names, and the locations of sample collection are indicated by the symbols according to the legend.

**Figure 2:**
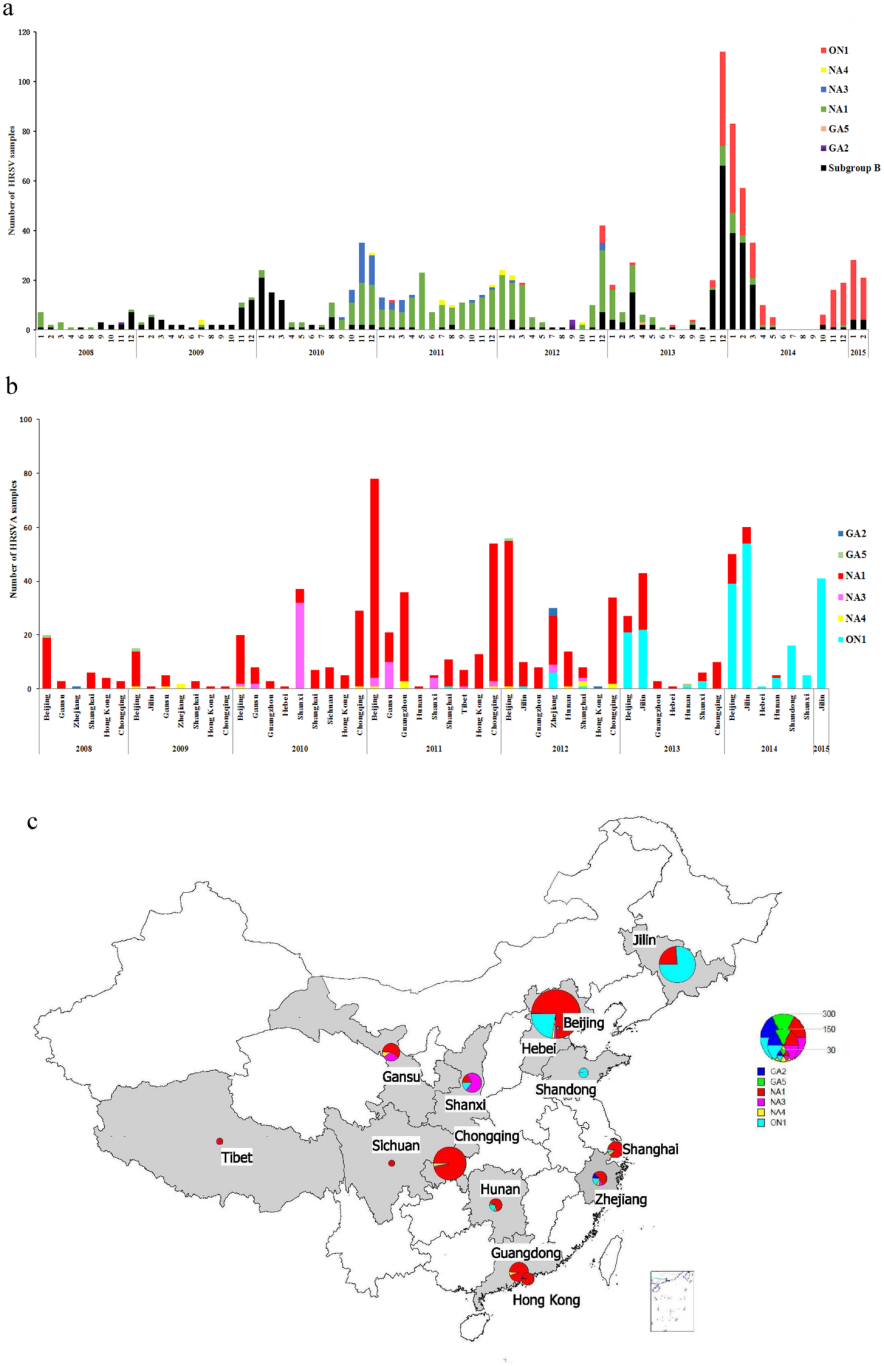
(**a**) Monthly distribution of HRSV subgroups and HRSVA genotypes between January 2008 and February 2015 in China. HRSVB subgroup and different genotypes of the HRSVA subgroup are colored according to the legend. (**b**) Yearly and geographic distribution of HRSVA genotypes from 2008 to 2015 in China. Different HRSVA genotypes are colored according to the legend. (**c**) Geographic distribution of HRSVA genotypes between January 2008 and February 2015 in China. The pie map of China was generated using MapInfo professional software (version 11.0, http://www.pitneybowes.com/us/location-intelligence/geographic-information-systems/mapinfo-pro.html). The number of the different genotypes is colored according to the legend. The 14 provinces where sequences were obtained are shaded in grey.

The HVR2 sequence of the G gene from 840 Chinese HRSVA sequences (337 sequences obtained in this study and 503 sequences downloaded from GenBank) were examined by molecular phylogenetic analysis. All HRSVA sequences clustered into 6 genotypes in the analysis, NA1 (539, 64.17%), ON1 (216, 25.71%), NA3 (59, 7.02%), NA4 (17, 2.02%), GA2 (5, 0.60%) and GA5 (4, 0.48%) (Fig. 1a). The NA1 genotype constituted the majority of the HRSVA sequences and most of the samples of this genotype were collected between 2008 and 2013, while the ON1 genotype was the next in abundance and most of the patient samples of this genotype were collected from 2013 to 2015 (Fig. 2a and b). A number of Chinese ON1 genotypes with lineage-specific substitutions as described below emerged as a potential new lineage from the ON1 genotype with 55% bootstrap value, and we have designated it as lineage 1 (Fig. 1a). The average nucleotide pairwise distance between the sequences from lineage 1 and the other sequences of the ON1 genotype was 0.029 ± 0.009 (mean ± standard deviation). The phylogenetic tree of all Chinese HRSVA sequences is shown in Fig. 1a and that of representative Chinese HRSVA sequences is shown in Fig. 1b. Representative Chinese sequences were chosen from sequences with pairwise distances > 0.025.

### Distribution of HRSV subgroups and HRSVA genotypes in China

The HRSV seasons are winter and spring in China, from October of one year to May of the next year. For the surveillance of HRSV infection between 2008 to 2015 in China, we found that HRSVA and HRSVB occasionally co-circulated in the same HRSV season, but more often, one predominant subgroup was observed in each season (Fig. 2a). HRSVB was the predominant subgroup in the 2008/2009 and 2009/2010 seasons, while HRSVA was prevalent in the 2010/2011 and 2011/2012 seasons. However, both HRSVA and HRSVB co-circulated in China from September 2012 to March 2014, whereas HRSVA subsequently became the predominant subgroup during the 2014/2015 season. Moreover, of all the sequences included in the analysis, 216 of them were ON1 genotype, which had a 72-nucleotide duplication in the sequence encoding the C-terminal of the G protein. The first ON1 genotype sample identified in this study was isolated in Shanghai province in February 2011. Following that, a total of 12 (5.56%) ON1 genotype sequences were identified from 2011 to 2013, and a total of 203 sequences (93.98%) of the ON1 genotype were detected in 2013/2014 and 2014/2015 (Fig. 2a,b and c).

For the yearly and geographic distribution of HRSVA genotypes in China from 2008 to 2015, the NA1 genotype was the predominant genotype found in Beijing, Gansu, Shanghai, Chongqing, and the Hong Kong special administrative region (SAR) between 2008 and 2012, and circulated in Jilin, Hebei, Shaanxi, Guangdong, Sichuan, Hunan, Zhejiang, and Tibet provinces between 2009 and 2013 (Fig. 2b and c). The ON1 genotype became more and more prevalent from November 2013 to February 2015 in many regions of China, including Jilin, Shaanxi, Beijing, Hunan, Hebei and Shandong provinces (Fig. 2b and c).

### Amino acid analysis

**Figure 3:**
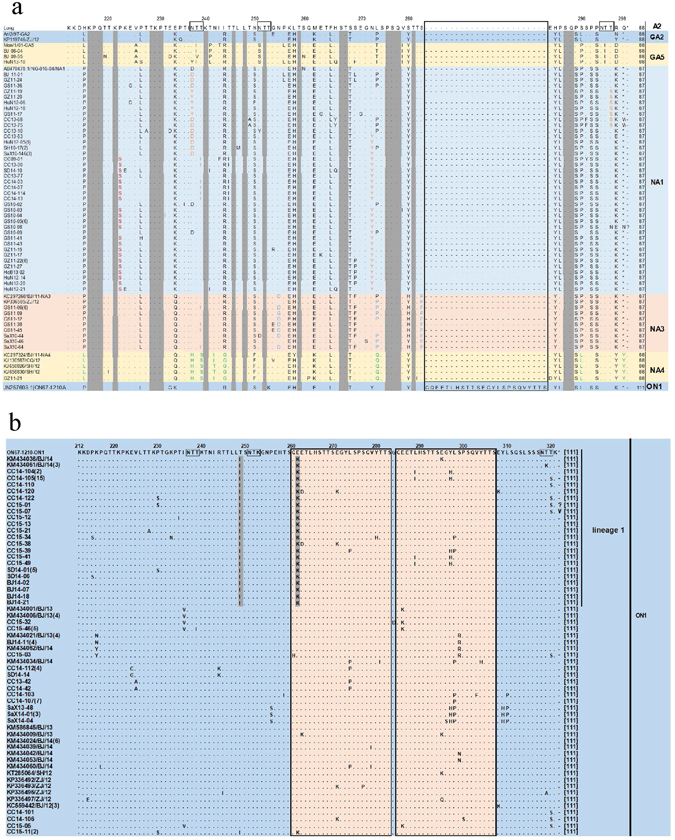
(**a**) Amino acid substitutions at the HVR2 region of the G protein in representative HRSVA sequences. Different background colors are used to facilitate visualization of different genotypes. Identical residues and stop codons are indicated by dots and asterisks, respectively; the lengths of the HVR2 region are shown next to the end of the sequences, followed by the genotype names. The regions shaded in grey represent the conserved regions among genotypes A2, GA2, GA5, NA1, NA3, NA4 and ON1. The box depicts the amino acid sequences corresponding to the 72-nucleotide duplication in the C-terminal of the G protein of the ON1 genotype. (**b**) Amino acid substitutions at the HVR2 region of the G protein in representative ON1 sequences. Some sequences clustered into lineage 1 due to the 2 substitutions of T249I and E262K, shaded in grey. The boxes depict the amino acid sequences corresponding to the 72-nucleotide duplication in the C-terminal of the G protein.

The predicted lengths of the G protein HVR2 regions of the HRSVA sequences analyzed in this study were 87 (genotypes NA1 and NA5), 88 (genotypes A2, GA2, GA5 and NA4) and 111 (genotype ON1) amino acids. For the 6 genotypes identified in this study, residues at amino acid positions 216–218, 221, 228–230, 240, 246, 248, 252, 259, 267–268, 276–278, 281 and 287–288 were highly conserved (Fig. 3a). The substitutions of S268T and P289S were found in the GA2, ON1, and all NA genotypes. When the Chinese ON1 sequences were compared with the sequence of the first ON1 genotype detected in Canada in 2010, (GenBank number: JN257693), 2 lineage-specific substitutions were identified in some of the Chinese ON1 genotypes: T249I and E262K; HRSVA with these substitutions clustered into a potential new lineage (lineage 1) within the ON1 genotype (Figs 1a and 3b). The E262K substitution occurred in the sequence duplication region in the HVR2 of ON1 (Fig. 3b).

## Discussion

In this study, the HVR2 regions of the G protein of 1471 Chinese HRSV samples (839 HRSVA, 631 HRSVB and 1 HRSVA/B) collected from 14 provinces between January 2008 and February 2015 were analyzed to determine the epidemiology of HRSV in China. All of the HRSVA sequences (337 sequences obtained in this study and 503 sequences downloaded from GenBank) were studied further for the distribution and sequence analysis of HRSVA. Hence, this study examined the molecular epidemiology and sequence characteristics of HRSVA circulating in China in recent years.

The monthly distribution of HRSV subgroups showed the alternating prevalence of HRSVA and HRSVB during the past 8 HRSV seasons in China. HRSVB was the predominant subgroup from 2008 to the spring of 2010, whereas HRSVA was the predominant subgroup in the following seasons from 2010 to 2012. Interestingly, HRSVB became more and more prevalent again starting at the end of 2012 until the spring of 2014 when both HRSVA and HRSVB co-circulated in China. The tendency of the replacement of the predominant subgroup indicated that alternating prevalence between HRSVA and HRSVB occurred every one or two years in China, which was also reported in other countries, such as Belgium and Finland^24, 25^.

Previous study showed GA2 was the predominant genotype of HRSVA circulating in some provinces in China from 2003/2004 to 2007/2008^5, 26–28^. However, the GA2 genotype was replaced by the NA1 genotype from 2008 to 2013 in China. The NA1 genotype was first reported in Beijing of China from 2007 to 2013^29^ and circulated in Chongqing between 2009 and 2013^30^. In this study, the NA1 genotype was also detected in Beijing and Chongqing, as well as in Gansu, Shanghai, Hong Kong SAR from 2008 to 2012 and expanded to circulate in other provinces, such as Jilin, Hebei, Shaanxi, Guangdong, Sichuan, Hunan, Zhejiang, and Tibet between 2009 and 2013. These data indicated that the NA1 genotype had circulated throughout mainland China from 2008 to 2013. The circulation of the NA1 genotype was also widely reported during the same HRSV seasons in Canada, Japan, Korea, Malaysia, and other countries^14, 18, 19, 31, 32^.

Similarly, the ON1 genotype was first detected in Canada in 2010, and its detection has been reported in the USA, Latvia, Germany, Cyprus, Kenya, China, Korea, Japan, Malaysia, Western India, Thailand and other countries since then^21^. In our study, the ON1 genotype was first detected in Shanghai in February 2011, which was earlier than its detection in Beijing in 2012 as reported previously^15, 29^. After that, the ON1 genotype became more and more prevalent, and replaced the NA1 genotype to become the predominant genotype in China between 2013 and 2015. Globally, the ON1 genotype has been reported in 21 countries and was evolving and disseminating quickly throughout the world with different ON1 lineages^21^. Based on the high prevalence and quick evolution of the ON1 genotype, it is expected that new lineages would emerge from the this genotype. In our study, we detected a potential new lineage (lineage 1) of the ON1 genotype in samples collected from 2014 to 2015 in China.

The sequence duplication in the ON1 genotype occurs in the HVR2 region of the G protein, an important antigenic region containing multiple epitopes. The structural flexibility of this region allows HRSV to generate variants with changes in key epitopes to overcome the pressure of antibodies raised after a natural infection^33^. In additional, the 24-amino acid duplication found in the ON1 genotype increases the length and/or change the structure of the G protein, which may contribute to the antigenicity of the G protein or evade host immunity. Interestingly, a similar sequence insertion was discovered in the HRSVB subgroup years before the discovery of the ON1 genotype: genotype BA with a 20-amino acid insertion in the HVR2 region of the G protein. More importantly, the BA genotype has circulated all over the world and evolved into 12 subgenotypes since its emergence in 1999^16, 34^. The spread of the BA genotype globally during the past decade suggested that the sequence repeat may provide some advantage to the virus^35^. Recent report showed that the BA genotype with the sequence duplication had a fitness advantage compared to the virus without the sequence duplication *in vitro*: the 20-amino acid insertion augmented virus attachment and fitness^36^. The ON1 genotype has circulated in many countries during the past 5 years since its emergence in 2010. Continuous molecular epidemiology surveillance needs to be conducted to determine if the sequence insertion also influences the transmissibility of the ON1 genotype and if the ON1 genotype circulates widely and for a long period of time like the BA genotype.

When compared with the first identified ON1 genotype sequence (Canada strain, ON67–1210, GenBank number: JN257693), a number of Chinese ON1 sequences identified in this study had 2 amino acid substitutions in the G protein: T249I and E262K. Sequences with these 2 substitutions clustered into a new lineage, named lineage 1 in this study. Both substitutions were detected in Beijing, Shandong and Jilin provinces in 2014 and 2015. The lineage 1 sequences from Beijing were classified as a new branch named ON1/NB previously^37^. The 3 provinces with lineage 1 sequences were located in northern and eastern China, where winter and spring last longer than most of the other parts of China. Studies had shown that for both HRSV subgroups, the geographical and temporal factors may play a role in the their genetic evolution^18, 38^. Although the impact of these 2 substitutions to the biologic function of the G protein was not clear to date, these 2 amino acids are located close to the antigenic sites^21^. Due to these substituions, the property of the amino acids changed from hydrophilic to hydrophobic for residue 249, and from acidic to alkaline for residue 262. Both changes might affect the antigenicity of the protein and/or viral attachment to the host. Residue 249 is close to the antigenic site at amino acids 250–258^39^, and this site has been suggested to be under positive selective pressure^40^. Residue 262 had also been suggested to undergo adaptive evolution^41^. Interesting, while substitution from Glu to Lys at residue 262 (E262K) was detected in samples from lineage 1 in the ON1 genoype, the same amino acid (Lys) is found at the same residue (262) in sequences of the GA2 and NA4 genotypes (i.e. K262). As shown in this study, the GA2 and NA4 genotypes had not circulated in China in recent years, while the NA1 genotype was found circulating widely in China from 2008 to 2013. The residue 262 of the G protein of the NA1 genotype was Glu, which was different from Met found at the same residue in the reference strain (Long). The biological significance of the substitution at residue 262 from Glu (e.g. NA1 and ON1 except lineage 1) to Lys (e.g. GA2, NA4 and lineage 1) or from Met (e.g. Long) to Glu (e.g. NA1 and ON1 except lineage 1) remains to be determined.

The genotype-specific substitutions identified in the NA3 genotype included S283P, as well as N255D and S280H which have been reported previously^42^. Substitution S283P was found in all of our NA3 sequences, whereas substitutions N255D and S280H occurred in most of the genotype NA3 sequences with the exception that 2 sequences had no substitutions at residue 255, but had S280Y instead of S280H. Meanwhile, the genotype NA4-specific substitutions N237H, T239S, I244G, and L274Q were found in all of the NA4 sequences in this study. Substitution N242I was identified as a genotype NA4-specific substitution from samples collected in Beijing previously^42^, but a mixure of 2 different substittuions were identified at residue 242 in NA4 sequences analyzed in this study: N242I (in a total of 3 sequences from Beijing, Guangzhou and Chongqing) and N242T (in 2 sequences from Shanghai). The significance of the genotype-specific substitutions requires further investigation.

Among the 337 HRSVA sequences generated in this study, 52 of them were from clinical isolates and the rest of them were from clinical samples that have not been passaged *in vitro*. We have compared these 52 sequences with the sequence of a reference strain (Long strain) and sequences from clinical samples. No specific amino acid changes were detected in sequences from clinical isolates; the substitutions detected in the sequences from these 52 clinical isolates were also found in sequences from clinical samples.

There were some limitations of our study due to the difference of sample collection in each year or geographic location that may impact the estimation of the prevalence of HRSVA genotypes in China: fewer samples were collected in 2008–2009, the number of samples from each province was different, and samples were not collected from some provinces every year. Also, samples were collected from 14 representative provinces instead of from all provinces in China. Due to the large amount of data generated from the analysis of Chinese HRSVA and HRSVB sequences in this project, results of the analysis for HRSVA and HRSVB sequences are described in separate reports.

In conclusion, this is an extensive study to investigate the yearly and geographic distribution of HRSV in 14 provinces in China from 2008 to 2015. After 5 years of consecutive circulation in mainland China from 2008 to 2012, the NA1 genotype appeared to have been replaced by the ON1 genotype in recent years. The ON1 genotype, detected as early as in 2011 in Shanghai, has become the predominant HRSVA genotype circulating in China between 2013 and 2015. Due to the widespread transmission and rapid evolution of HRSV, continuous and long-term epidemiological surveillance and molecular characterization need to be conducted to provide the scientific basis for the prevention and treatment of HRSV infection in the future.

## Methods

### Ethics statement

In this study, there was no human experimentation; only nasopharyngeal aspirates or nasopharyngeal precipitates were collected from patients who were identified as potentially infected with HRSV infection. Written informed consent for the use of clinical specimens was obtained from each patient involved in this study. This study was approved by the second session of the Ethics Review Committee of the National Institute for Viral Disease Control and Prevention in the Chinese Center for Disease Control and Prevention (CDC) and the methods were conducted according to the guidelines.

### Sample collection

4246 nasopharyngeal aspirates or nasopharyngeal precipitates were collected from patients who were hospitalized with acute respiratory illness or outpatients who had the symptoms of acute respiratory infections, such as fever, cough, dyspnea and other symptoms from 9 sentinel hospitals in 9 provinces in China, including Jilin, Beijing, Hebei, Gansu, Shaanxi, Shanghai, Hunan, Guangdong and Shandong provinces, respectively, from 2008 to 2015. Some clinical samples were inoculated into Hep-2 cells for virus isolation. The culture supernatants and the clinical samples were frozen and stored at −80 0C for further analysis.

### RNA extraction and subgroup screening

Total RNA from clinical isolates or clinical samples were extracted using the QIAamp DNA/RNA mini kit (QIAGEN, Valencia, CA, USA, CAT: 52906 or 74106) according to the manufacturer’s instructions. Double channel real-time RT-PCR was used for HRSV detection, as well as HRSVA and HRSVB subgroup identification^23^.

### Nucleotide amplification and sequence determination for HRSVA

The partial sequence of the G gene of HRSVA samples (634–897 nt) were amplified by RT-PCR using the One Step RT-PCR kit (TaKaRa Biotechnology Dalian, China, cat: DRR057A) with the primer pairs described previously^26, 43^. Primer sets GPA-F1 were used to amplify and sequence the nucleotides encoding the HVR2 region located at the C-terminal region of the G protein of HRSVA samples. The HRSVA PCR products were purified and sequenced using an ABI Prism 3710xl DNA Analyzer. The sequences of HRSVA were edited and aligned using Sequencher software verion 5.0 (Gene Codes, Ann Arbor, MI, USA).

The sequences generated in this study were submitted to GenBank with the accession numbers from KX533544 to KX533879.

### Other HRSV sequences used in this study

All (766) Chinese HRSV sequences from samples collected from 2008 to 2015 that were available from GenBank on February 2016, as well as the reference sequences of different HRSVA genotypes were downloaded from GenBank. The HVR2 regions of HRSVA G protein sequences were aligned using the software MEGA software (version 5.0). Information of the sequences downloaded from GenBank was showed in the Supplementary Table 1.

### Phylogenetic analysis and pairwise distance calculation

Alignment and phylogenetic analysis of the sequences were conducted using the software MEGA 5.0^44^. The phylogenetic tree was generated using the neighbor-joining method and the Kimura 2-parameter method was chosen for substitution model with pairwise deletion treatment for the missing and gaps data. Evaluation of the reliability of phylogenetic inference was estimated using the bootstrap method with 1000 replicates with a cut-off of usually ≥ 75% in MEGA 5.0 program. Pairwise distance between different strains was also calculated by MEGA 5.0 as previously described^45–47^.

### Statistical Analysis

Differences in clinical characteristic of HRSV infection between males and females, as well as between 2 different age groups were calculated using the SPSS17.0 software with Pearson Chi-Square test, and P-value < 0.05 was considered to be statistically significant.

## Electronic supplementary material

supplementary Table 1

## References

[1] Wu A (2015). Incidence and Risk Factors for Respiratory Syncytial Virus and Human Metapneumovirus Infections among Children in the Remote Highlands of Peru. PLoS One.

[2] Pretorius MA (2016). The role of influenza, RSV and other common respiratory viruses in severe acute respiratory infections and influenza-like illness in a population with a high HIV sero-prevalence, South Africa 2012–2015. J Clin Virol.

[3] Turner TL (2014). Respiratory syncytial virus: current and emerging treatment options. Clinicoecon Outcomes Res.

[4] Liu T (2015). Viral Etiology of acute respiratory tract infections in hospitalized children and adults in Shandong Province, China. Virol J.

[5] Zhang RF (2010). Human respiratory syncytial virus in children with acute respiratory tract infections in China. J Clin Microbiol.

[6] Resch B (2012). Burden of respiratory syncytial virus infection in young children. World J Clin Pediatr.

[7] Feng L (2014). Viral etiologies of hospitalized acute lower respiratory infection patients in China, 2009–2013. PLoS One.

[8] Goldstein E, Greene SK, Olson DR, Hanage WP, Lipsitch M (2015). Estimating the hospitalization burden associated with influenza and respiratory syncytial virus in New York City, 2003-2011. Influenza Other Respir Viruses.

[9] Branche AR, Falsey AR (2015). Respiratory syncytial virus infection in older adults: an under-recognized problem. Drugs Aging.

[10] Cane PA (2001). Molecular epidemiology of respiratory syncytial virus. Rev Med Virol.

[11] Mufson MA, Orvell C, Rafnar B, Norrby E (1985). Two distinct subtypes of human respiratory syncytial virus. J Gen Virol.

[12] Avadhanula V (2015). Infection with novel respiratory syncytial virus genotype Ontario (ON1) in adult hematopoietic cell transplant recipients, Texas, 2011-2013. J Infect Dis.

[13] Martinelli M (2014). Phylogeny and population dynamics of respiratory syncytial virus (Rsv) A and B. Virus Res.

[14] Tsukagoshi H (2013). Genetic analysis of attachment glycoprotein (G) gene in new genotype ON1 of human respiratory syncytial virus detected in Japan. Microbiol Immunol.

[15] Liu J (2014). Genetic variation of human respiratory syncytial virus among children with fever and respiratory symptoms in Shanghai, China, from 2009 to 2012. Infect Genet Evol.

[16] Ren L, Xiao Q, Zhou L, Xia Q, Liu E (2015). Molecular characterization of human respiratory syncytial virus subtype B: a novel genotype of subtype B circulating in China. J Med Virol.

[17] Dapat IC (2010). New genotypes within respiratory syncytial virus group B genotype BA in Niigata, Japan. J Clin Microbiol.

[18] Eshaghi A (2012). Genetic variability of human respiratory syncytial virus A strains circulating in Ontario: a novel genotype with a 72 nucleotide G gene duplication. PLoS One.

[19] Baek YH (2012). Prevalence and genetic characterization of respiratory syncytial virus (RSV) in hospitalized children in Korea. Arch Virol.

[20] Blanc A, Delfraro A, Frabasile S, Arbiza J (2005). Genotypes of respiratory syncytial virus group B identified in Uruguay. Arch Virol.

[21] Duvvuri VR (2015). Genetic diversity and evolutionary insights of respiratory syncytial virus A ON1 genotype: global and local transmission dynamics. Sci Rep.

[22] Hirano E (2014). Molecular evolution of human respiratory syncytial virus attachment glycoprotein (G) gene of new genotype ON1 and ancestor NA1. Infect Genet Evol.

[23] van Elden LJ (2003). Applicability of a real-time quantitative PCR assay for diagnosis of respiratory syncytial virus infection in immunocompromised adults. J Clin Microbiol.

[24] Zlateva KT, Vijgen L, Dekeersmaeker N, Naranjo C, Van Ranst M (2007). Subgroup prevalence and genotype circulation patterns of human respiratory syncytial virus in Belgium during ten successive epidemic seasons. J Clin Microbiol.

[25] Waris M (1991). Pattern of respiratory syncytial virus epidemics in Finland: two-year cycles with alternating prevalence of groups A and B. J Infect Dis.

[26] Zhang Y (2007). Genetic variability of group A and B human respiratory syncytial viruses isolated from 3 provinces in China. Arch Virol.

[27] Mao, H., Lu,Y. & Yan, J. Genetic Characteristics of the G Gene of RSV in Zhejiang Province. *Virol Sin* **21**, 111–115 (2006).

[28] Zhang, Y. *et al*. Study on Genetic Diversity of the G Protein of Subgroups A and B Human Respiratory Syncytial Viruses Isolated in Beijing, China. *Chin J Virol* **21**, 332–342 (2005).

[29] Cui G (2013). Emerging human respiratory syncytial virus genotype ON1 found in infants with pneumonia in Beijing, China. Emerg Microbes Infect.

[30] Ren L (2014). The genetic variability of glycoproteins among respiratory syncytial virus subtype A in China between 2009 and 2013. Infect Genet Evol.

[31] Etemadi MR, Sekawi Z, Othman N, Lye MS, Moghaddam FY (2013). Circulation of human respiratory syncytial virus strains among hospitalized children with acute lower respiratory infection in malaysia. Evol Bioinform Online.

[32] Khor CS, Sam IC, Hooi PS, Chan YF (2013). Displacement of predominant respiratory syncytial virus genotypes in Malaysia between 1989 and 2011. Infect Genet Evol.

[33] Melero JA, Garcia-Barreno B, Martinez I, Pringle CR, Cane PA (1997). Antigenic structure, evolution and immunobiology of human respiratory syncytial virus attachment (G) protein. J Gen Virol.

[34] Trento A (2003). Major changes in the G protein of human respiratory syncytial virus isolates introduced by a duplication of 60 nucleotides. J Gen Virol.

[35] McLellan JS, Ray WC, Peeples ME (2013). Structure and function of respiratory syncytial virus surface glycoproteins. Curr Top Microbiol Immunol.

[36] Hotard AL, Laikhter E, Brooks K, Hartert TV, Moore ML (2015). Functional Analysis of the 60-Nucleotide Duplication in the Respiratory Syncytial Virus Buenos Aires Strain Attachment Glycoprotein. J Virol.

[37] Cui, G. *et al*. Rapid replacement of prevailing genotype of human respiratory syncytial virus by genotype ON1 in Beijing, 2012–2014. *Infect Genet Evol* **33**, 163–8 (2015). 10.1016/j.meegid.2015.04.025 25929164

[38] Kuroiwa Y (2005). A phylogenetic study of human respiratory syncytial viruses group A and B strains isolated in two cities in Japan from 1980–2002. J Med Virol.

[39] Cane PA (1997). Analysis of linear epitopes recognised by the primary human antibody response to a variable region of the attachment (G) protein of respiratory syncytial virus. J Med Virol.

[40] Botosso VF (2009). Positive selection results in frequent reversible amino acid replacements in the G protein gene of human respiratory syncytial virus. PLoS Pathog.

[41] Zlateva KT, Lemey P, Vandamme AM, Van Ranst M (2004). Molecular Evolution and Circulation Patterns of Human Respiratory Syncytial Virus Subgroup A: Positively Selected Sites in the Attachment G Glycoprotein. Journal of Virology.

[42] Cui G (2013). Genetic variation in attachment glycoprotein genes of human respiratory syncytial virus subgroups a and B in children in recent five consecutive years. PLoS One.

[43] Peret TC, Hall CB, Schnabel KC, Golub JA, Anderson LJ (1998). Circulation patterns of genetically distinct group A and B strains of human respiratory syncytial virus in a community. J Gen Virol.

[44] Tamura K (2011). MEGA5: molecular evolutionary genetics analysis using maximum likelihood, evolutionary distance, and maximum parsimony methods. Mol Biol Evol.

[45] Saitou N, Nei M (1987). The neighbor-joining method: a new method for reconstructing phylogenetic trees. Mol Biol Evol.

[46] Zharkikh A, Li WH (1995). Estimation of confidence in phylogeny: the complete-and-partial bootstrap technique. Mol Phylogenet Evol.

[47] Kimura M (1980). A simple method for estimating evolutionary rates of base substitutions through comparative studies of nucleotide sequences. J Mol Evol.

