# Supplementary material for: Emergence of ON1 genotype of human respiratory syncytial virus subgroup A in China between 2011 and 2015
Source: Sci Rep. 2017 Jul 14;7:5501. doi: 10.1038/s41598-017-04824-0 (PMC5511225; doi:10.1038/s41598-017-04824-0)
Supplement: Supplementary file 1 — supplementary Table 1 [file 41598_2017_4824_MOESM1_ESM.pdf]

Emergence of ON1 genotype of human respiratory syncytial virus subgroup A in China between 2011 and 2015

Jinhua Song[1*], Yan Zhang[1*], Huiling Wang[1], Jing Shi[1,2], Liwei Sun[3],Xiaojie Zhang[3], Zifeng Yang[4], Wenda Guan[4], Hong Zhang[5], Pengbo Yu[6], Zhengde Xie[7], Aili Cui[1], Teresa I. Ng[8] , Wenbo Xu[1,9#]

[1]WHO WPRO Regional Reference Measles/Rubella Laboratory and Key Laboratory of Medical Virology Ministry of Health, National Institute for Viral Disease Control and Prevention, China Center for Disease Control and Prevention, Beijing, People's Republic of China, [2]Lu Juan Community Health Center of Daxing Region, Beijing, People's Republic of China, [3]Jilin Children's Medical Center, Children's Hospital of Changchun, Changchun, People's Republic of China, [4]State Key Laboratory of Respiratory Disease, National Clinical Research Center for Respiratory Disease, First Affiliated Hospital of Guangzhou Medical University, Guangzhou, Guangdong, People's Republic of China, [5]Hunan Provincial Centers for Disease Control and Prevention, Changsha, People's Republic of China, [6]Shaanxi Provincial Centers for Disease Control and Prevention, Xian, People's Republic of China, [7]Beijing Children's Hospital, Capital Medical University, Beijing, People's Republic of China, [8]AbbVie, Inc., North Chicago, IL, USA. [9]Medical College, Anhui University of Science & Technology, Huainan, People's Republic of China

*These authors contributed equally to this work.

[#]Corresponding author: Wenbo Xu, E-mail address:wenbo_xu1@aliyun.com.

Supplementary Table 1. Accession numbers of Chinese HRSV sequences downloaded from GenBank for analysis

Accession No.	HRSV Subgroup
KT285064	A
KP336492	A
KP336493	A
KP336494	A
KP336495	A
KP336496	A
KP336497	A
KP336498	A
KP336499	A
KP336500	A
KP336501	A
KP336502	A
KP336503	A
KP336504	A
KP336505	A
KP336506	A
KP336507	A
KP336512	A
KP336513	A
KP336514	A
KP336515	A
KP336516	A
KP336517	A
KP336518	A
KP336519	A
KP336520	A
KP336521	A
KP336522	A
GU357541	A
GU357542	A
GU357543	A
GU357544	A
GU357545	A
GU357546	A
GU357547	A

GU357548	A
JQ322971	A
JQ322972	A
JQ322973	A
JQ322974	A
JQ322975	A
JQ322976	A
JQ322977	A
GU550471	A
GU550472	A
GU550473	A
JF713447	A
JF713448	A
JF713449	A
JF713450	A
JF713451	A
JF713452	A
JF713453	A
JF713454	A
JN968299	A
JN968300	A
JN968301	A
JN968302	A
JN968303	A
JN968304	A
JN968305	A
JN968306	A
JN968307	A
JN968308	A
JN968309	A
JN968310	A
JN968311	A
JN968313	A
JN968323	A
JN968324	A
JN968325	A
JN968326	A
JN968327	A
JN968328	A

JN968329	A
JN968330	A
JN968331	A
JX885113	A
JX885112	A
JX885121	A
JX885122	A
JX885123	A
JX885125	A
JX885127	A
JX885128	A
JX885129	A
JX885130	A
KC297233	A
KC297234	A
KC297235	A
KC297236	A
KC297237	A
KC297238	A
KC297239	A
KC297240	A
KC297241	A
KC297242	A
KC297243	A
KC297244	A
KC297245	A
KC297246	A
KC297247	A
KC297248	A
KC297249	A
KC297250	A
KC297251	A
KC297252	A
KC297253	A
KC297254	A
KC297255	A
KC297256	A
KC297257	A
KC297258	A

KC297259	A
KC297260	A
KC297261	A
KC297262	A
KC297263	A
KC297264	A
KC297265	A
KC297266	A
KC297267	A
KC297268	A
KC297269	A
KC297270	A
KC297271	A
KC297272	A
KC297273	A
KC297274	A
KC297275	A
KC297276	A
KC297277	A
KC297278	A
KC297279	A
KC297280	A
KC297281	A
KC297282	A
KC297283	A
KC297284	A
KC297285	A
KC297286	A
KC297287	A
KC297288	A
KC297289	A
KC297290	A
KC297291	A
KC297292	A
KC297293	A
KC297294	A
KC297295	A
KC297296	A
KC297297	A

KC297298	A
KC297299	A
KC297300	A
KC297301	A
KC297302	A
KC297303	A
KC297304	A
KC297305	A
KC297306	A
KC297307	A
KC297308	A
KC297309	A
KC297310	A
KC297311	A
KC297312	A
KC297313	A
KC297314	A
KC297315	A
KC297316	A
KC297317	A
KC297318	A
KC297319	A
KC297320	A
KC297321	A
KC297322	A
KC297323	A
KC297324	A
KC297325	A
KC297326	A
KC297327	A
KC297328	A
KC297329	A
KC297330	A
KC297331	A
KC297332	A
KC297333	A
KC297334	A
KC297335	A
KC297336	A

KC297337	A
KC297338	A
KC297339	A
KC297340	A
KC297341	A
KC297342	A
KC297343	A
KC297344	A
KC297345	A
KC297346	A
KC297347	A
KC297348	A
KC297349	A
KC297350	A
KC297351	A
KC297352	A
KC297353	A
KC297354	A
KC297355	A
KC297356	A
KC297357	A
KC297358	A
KC297359	A
KC297360	A
KC297361	A
KC297362	A
KC297363	A
KC297364	A
KC297365	A
KC297366	A
KC297367	A
KC297368	A
KC297369	A
KC297370	A
KC297371	A
KC297372	A
KC297373	A
KC297374	A
KC297375	A

KC297376	A
KC297377	A
KC297378	A
KC297403	A
KC297404	A
KC297405	A
KC297406	A
KC297407	A
KC297408	A
KC297409	A
KC297410	A
KC297411	A
KC297412	A
KC297413	A
KC297414	A
KC297415	A
KC297416	A
KC297417	A
KC297418	A
KC297419	A
KC297420	A
KC297421	A
KC461268	A
KC461269	A
KC461270	A
KC461271	A
KC461272	A
KC461273	A
KC461274	A
KC461275	A
KC461276	A
KC461277	A
KC461278	A
KC461279	A
KC461280	A
KC461281	A
KC461282	A
KC461283	A
KC461284	A

KC461285	A
KC461286	A
KC461287	A
KC559440	A
KC559442	A
KC461212	A
KC461213	A
KC559441	A
KC559443	A
KC559444	A
KC559445	A
KC559446	A
KC559447	A
KC559448	A
KC978856	A
KJ130547	A
KJ130548	A
KJ130549	A
KJ130550	A
KJ130551	A
KJ130552	A
KJ130553	A
KJ130554	A
KJ130555	A
KJ130556	A
KJ130557	A
KJ130558	A
KJ130559	A
KJ130560	A
KJ130561	A
KJ130562	A
KJ130563	A
KJ130564	A
KJ130565	A
KJ130566	A
KJ130567	A
KJ130568	A
KJ130569	A
KJ130570	A

KJ130571	A
KJ130572	A
KJ130573	A
KJ130574	A
KJ130575	A
KJ130576	A
KJ130577	A
KJ130578	A
KJ130579	A
KJ130580	A
KJ130581	A
KJ130582	A
KJ130583	A
KJ130584	A
KJ130585	A
KJ130586	A
KJ130587	A
KJ130588	A
KJ130589	A
KJ130590	A
KJ130591	A
KJ130592	A
KJ130593	A
KJ130594	A
KJ130595	A
KJ130596	A
KJ130597	A
KJ130598	A
KJ130599	A
KJ130600	A
KJ130601	A
KJ130602	A
KJ130603	A
KJ130604	A
KJ130605	A
KJ130606	A
KJ130607	A
KJ130608	A
KJ130609	A

KJ130610	A
KJ130611	A
KJ130612	A
KJ130613	A
KJ130614	A
KJ130615	A
KJ130616	A
KJ130617	A
KJ130618	A
KJ130619	A
KJ130620	A
KJ130621	A
KJ130622	A
KJ130623	A
KJ130624	A
KJ130625	A
KJ130626	A
KJ130627	A
KJ130628	A
KJ130629	A
KJ130630	A
KJ130631	A
KJ130632	A
KJ130633	A
KJ130634	A
KJ130635	A
KJ130636	A
KJ130637	A
KJ130638	A
KJ130639	A
KJ130640	A
KJ130641	A
KJ130642	A
KJ130643	A
KJ130644	A
KJ658811	A
KJ658812	A
KJ658813	A
KJ658814	A

KJ658815	A
KJ658816	A
KJ658817	A
KJ658818	A
KJ658819	A
KJ658820	A
KJ658821	A
KJ658822	A
KJ658823	A
KJ658824	A
KJ658825	A
KJ658826	A
KJ658827	A
KJ658828	A
KJ658829	A
KJ658830	A
KJ658831	A
KJ658832	A
KM433999	A
KM434000	A
KM434001	A
KM434002	A
KM434003	A
KM434004	A
KM434014	A
KM434005	A
KM434006	A
KM434007	A
KM434008	A
KM434009	A
KM434010	A
KM434011	A
KM434012	A
KM434013	A
KM434015	A
KM434016	A
KM434017	A
KM434018	A
KM434019	A

KM434021	A
KM434022	A
KM434024	A
KM434025	A
KM434026	A
KM434030	A
KM434033	A
KM434034	A
KM434036	A
KM434037	A
KM434039	A
KM434040	A
KM434041	A
KM434042	A
KM434043	A
KM434044	A
KM434045	A
KM434046	A
KM434049	A
KM434050	A
KM434051	A
KM434053	A
KM434056	A
KM434057	A
KM434058	A
KM434060	A
KM434061	A
KM434062	A
KM586834	A
KM586845	A
KM586837	A
KM434027	A
KM434029	A
KM434031	A
KM434032	A
KM434035	A
KM434038	A
KM434023	A
KM434020	A

KM434047	A
KM434048	A
KM434052	A
KM434055	A
KM434059	A
KM517572	A
KM578843	A
KM586819	A
KM586820	A
KM586821	A
KM586822	A
KM586823	A
KM586824	A
KM586825	A
KM586826	A
KM586827	A
KM586828	A
KM586829	A
KM586830	A
KM586831	A
KM586832	A
KM586833	A
KM586839	A
KM586841	A
KM586842	A
KP119747	A
KP119745	A
KP119746	A
KP119748	A
KP218910	A
KR607995	A
KR607996	A
KM517573	B
KP336523	B
KP336524	B
KP336525	B
KP336526	B
KP336527	B
KP336528	B

KP336529	B
KP336530	B
KP336531	B
KP336532	B
KP336533	B
KP336534	B
KP336535	B
KP336536	B
KP336537	B
KP336538	B
KP336539	B
KP336540	B
KP336541	B
KP336542	B
KP336543	B
KP336544	B
KP336545	B
KP336546	B
GU357503	B
GU357504	B
GU357505	B
GU357508	B
GU357509	B
GU357510	B
GU357511	B
GU357512	B
GU357514	B
GU357515	B
GU357516	B
GU357520	B
GU357521	B
GU357523	B
GU357524	B
GU357525	B
GU357526	B
GU357527	B
GU357528	B
GU357529	B
GU357530	B

GU550479	B
GU550480	B
GU550482	B
GU550483	B
GU550484	B
GU550485	B
GU550486	B
GU550487	B
GU550488	B
GU550490	B
GU550491	B
GU550492	B
GU550493	B
GU550494	B
GU550495	B
GU550496	B
GU550498	B
GU550501	B
GU550502	B
GU550503	B
JF713439	B
JF713440	B
JF713442	B
JF713443	B
JN968345	B
JN968346	B
JN968350	B
JN968351	B
JN968352	B
JN968353	B
JN968354	B
JN968355	B
JN968356	B
JN968358	B
JN968359	B
JN968362	B
JN968363	B
JX885106	B
JX885107	B

JX885108	B
JX885109	B
JX885110	B
JX885111	B
JX885114	B
JX885115	B
JX885116	B
JX885117	B
JX885118	B
JX885119	B
JX885120	B
JX885126	B
JX885124	B
KC297422	B
KC297423	B
KC297424	B
KC297425	B
KC297426	B
KC297429	B
KC297431	B
KC297433	B
KC297434	B
KC297435	B
KC297436	B
KC297437	B
KC297438	B
KC297439	B
KC297440	B
KC297441	B
KC297442	B
KC297443	B
KC297444	B
KC297445	B
KC297447	B
KC297448	B
KC297449	B
KC297452	B
KC297453	B
KC297455	B

KC297456	B
KC297457	B
KC297458	B
KC297459	B
KC297460	B
KC297461	B
KC297463	B
KC297464	B
KC297465	B
KC297467	B
KC297469	B
KC297473	B
KC297475	B
KC297476	B
KC297477	B
KC297481	B
KC297482	B
KC297483	B
KC297484	B
KC297485	B
KC297486	B
KC297487	B
KC297488	B
KC297489	B
KC297490	B
KC297491	B
KC297492	B
KC297493	B
KC461262	B
KC461263	B
KC461264	B
KC461265	B
KC461266	B
KC461267	B
KM586835	B
KM586836	B
KM586838	B
KM586840	B
KM586843	B

KM586844	B
KR607978	B
KR607979	B
KR607980	B
KR607981	B
KR607982	B
KR607983	B
KR607984	B
KJ658777	B
KJ658778	B
KJ658779	B
KJ658780	B
KJ658781	B
KJ658782	B
KJ658783	B
KJ658784	B
KJ658785	B
KJ658786	B
KJ658787	B
KJ658788	B
KJ658789	B
KJ658790	B
KJ658791	B
KJ658792	B
KJ658793	B
KJ658794	B
KJ658795	B
KJ658796	B
KJ658797	B
KJ658798	B
KJ658799	B
KJ658800	B
KJ658801	B
KJ658802	B
KJ658803	B
KJ658804	B
KJ658805	B
KJ658806	B
KJ658807	B

KJ658808	B
KJ658809	B
KJ658810	B
KJ658764	B
KJ658765	B
KJ658766	B
KJ658767	B
KJ658768	B
KJ658769	B
KJ658770	B
KJ658771	B
KJ658772	B
KJ658773	B
KJ658774	B
KJ658775	B
GU550499	B
GU550500	B
GU550497	B
GU550489	B
GU550481	B
JF713441	B
GU357517	B
GU357518	B
GU357522	B
GU357519	B
GU357506	B
GU357507	B
KP336508	B
KP336509	B
KP336510	B
KP336511	B
GU357513	B
KC297450	B
KC297427	B
KC297428	B
KC461288	B
KC461289	B
KC461290	B
KC461291	B

KC461292	B
KC461293	B
KC461294	B
KC461295	B
KC297446	B
KC297454	B
KC297462	B
KC297451	B
KC297466	B
KC297468	B
KC297470	B
KC297471	B
KC297472	B
KC297474	B
KC297432	B
KC297430	B
JF713444	B
JF713445	B
JF713446	B
JN968293	B
JN968295	B
JN968343	B
KJ658776	A
GU068539	A
GU068540	A
GU068541	A
GU068542	A
GU068543	A
GU068544	A
